# NO-Donor Iron Nitrosyl Complex with *N*-Ethylthiourea Ligand Exhibits Selective Toxicity to Glioma A172 Cells

**DOI:** 10.3390/molecules22091426

**Published:** 2017-08-29

**Authors:** Nataliya Sanina, Natal’ya Shmatko, Tatiyana Stupina, Anastasiya Balakina, Alexei Terent’ev

**Affiliations:** 1 Institute of Problems of Chemical Physics RAS, Chernogolovka 142432, Russia; green4natasha@mail.ru (N.S.); stupina.tat@gmail.com (T.S.); stasya.balakina@gmail.com (A.B.); alexei@icp.ac.ru (A.T.); 2 Medicinal Chemistry Research and Education Center, Moscow Region State University, Moscow 105005, Russia; 3 Faculty of Fundamental Physical and Chemical Engineering, M.V. Lomonosov Moscow State University, Moscow 119991, Russia

**Keywords:** nitric oxide, NO donor, iron nitrosyl complex, *N*-ethylthiourea, cytotoxicity, glioma

## Abstract

We studied effects of NO-donor iron nitrosyl complex with *N*-ethylthiourea ligand (ETM) on normal or tumor-derived cell lines. ETM was mildly toxic to most cell lines studied except the human glioma cell line A172 that proved to be highly sensitive to the complex and underwent cell death after ETM exposure. The high susceptibility of A172 cells to ETM was attributed to its NO-donor properties since no toxicity was detected for the *N*-ethylthiourea ligand.

## 1. Introduction

Nitric oxide (NO) is an important signaling molecule involved in modulating diverse physiological processes such as vasodilatation, immune response, inflammation, and neurotransmission. It has also been implicated in many disorders, including cardiovascular, neurodegenerative, autoimmune diseases, and cancer [1].

Roles played by nitric oxide in cancer have been being studied extensively. It is well established that NO can possess both pro- and anti-tumorigenic activities which reflects its multifaceted functions. At nanomolar concentrations, NO functions as a signal transducer modifying activity of enzymes, e.g., guanilyl cyclase, but at moderate concentrations ranging from appr. 100 nM to several hundred nM, it induces different, and often opposing, effects, e.g., proliferation or cell cycle arrest, and, finally, at micromolar concentrations it induces nitrosative stress resulting in cell damage. Thus, functions of NO are differentially regulated by its concentrations [2], and it may be involved in tumor promotion, progression and metastasis, regulation of cell proliferation, cell death, angiogenesis, epithelial–mesenchymal transition, extravasation and invasion [3,4].

Regardless of the dual role of NO in tumors, a promising approach to cancer treatment is a NO donor based therapy. Although most studies concerning NO donors as antitumor agents have been performed in vitro, there are accumulating data from in vivo experiments and clinical trials demonstrating potential of NO donors for cancer therapy [5,6]. The major classes of NO-donor compounds comprise organic nitrates; metal-NO complexes, *S*-nitrosothiols, sydnonimines, diazeniumdiolates and NO-drug hybrids [7].

Dinitrosyl iron complexes (DNICs) constitute a class of metal-NO complexes that are closely related to NO functions. DNICs possess two NO groups and two thiolate ligands; the former can be thiol groups of proteins (protein-bound, or high molecular weight DNICs) or free thiols (low molecular weight DNICs). DNICs, together with *S*-nitrosothiols, are thought to be storage and transport forms of NO [8,9,10]. At the same time, DNICs are NO donors that decompose through non-enzymatic mechanism. At neutral pH, decomposition of DNICs results in the release of NO and a thiol [11,12].

First, synthetic DNICs as NO donors in biological systems were studied for their vasodilator ability in 1990s [13,14]. Later, DNICs were shown to induce the SOS response in bacteria [15,16].

Concerning cancer therapy, there is a growing interest in DNICs as potential antitumor NO donors. Thiosulfate-containing DNIC has been shown to induce caspase-independent apoptosis in the human Jurkat leukemia cell line [17]. The complex [K-18-crown-6-ether][S_5_Fe(NO)_2_] that combines crown ester and DNIC with sulfur rings as thiolate moieties was shown to be able to serve as a photochemical NO donor. In combination with UV-A light exposure, the complex greatly decreased the viability of human erythroleukemia K562 cells compared to the effects of UV-A or the complex alone [18]. DNICs with *N*,*N*-dimethyl-2-mercaptoacetamide ligand in different conformations and DNIC with 2-mercaptoethanol inhibited the viability of mouse melanoma B16-F10 cells [19,20]. DNICs with glutathione or thiosulfate ligands induced cell death in HeLa cells in the presence of chelating agents [21].

The antitumor activity of DNIC with mixed cysteamine/2-mercaptoethanol ligands was exhaustively studied by Wu and co-workers. The complex was shown to be untaken by cells, where it decomposes within 3 h, and the NO production was detected in 30 min post-exposure. The complex was toxic to tumor cell lines PC-3 (human prostate carcinoma), SKBR-3 (human breast carcinoma), and CRL5866 (human lung carcinoma); the IC_50_ values ranged from appr. 20 μM to 40 μM and its effects on the cells were accompanied by both activation of pro-apoptotic proteins and down-regulation of anti-apoptotic proteins. In vivo studies on nude mice bearing subcutaneous PC-3 tumor xenografts revealed that the complex can efficiently inhibit tumor growth after intravenous administration; no significant changes in the body weight, blood biochemical parameters and histology of tissues. Apoptosis and up-regulation of pro-apoptotic proteins in PC-3 tumor xenografts were also observed with immunohistochemical staining [22].

Over the past few years, we have synthesized a wide range of DNICs and Roussin’s Red Esters (RRE, the “dimeric” form of DNIC) and studies their antiproliferative effects in vitro. Dimeric DNIC with thiophenol ligands exerted similar toxicity to tumor HeLa (human cervical carcinoma) and H1299 (human non-small cell lung carcinoma) cells, and induced cell death with PARP degradation as an apoptotic sign [23]. RRE with 2-aminothiophenol ligand suppressed viability of SKOV3 (human ovarian carcinoma), LS174T (human colon carcinoma), MCF7 (human breast carcinoma), and A549 (human non-small cell lung carcinoma) cells. In experiments on mice bearing Ca-755 mammary carcinoma or B16 melanoma, when administered intraperitoneally, the complex exhibited moderate, but statistically significant, antitumor effects [24]. RRE with cysteamine ligand have been shown to inhibit proliferation of HeLa cells with relatively low IC_50_ values compared to other studied complexes. Both thiophenol- and cysteamine-bearing RREs disturbed the nuclear translocation and DNA binding of the nuclear factor NF-κB. In isolated nuclear extracts, RRE with cysteamine caused *S*-nitrosylation of the p65 subunit of NF-κB, which was proposed to be a molecular basis of antiproliferative effect of the complex [25].

**Figure 1:**
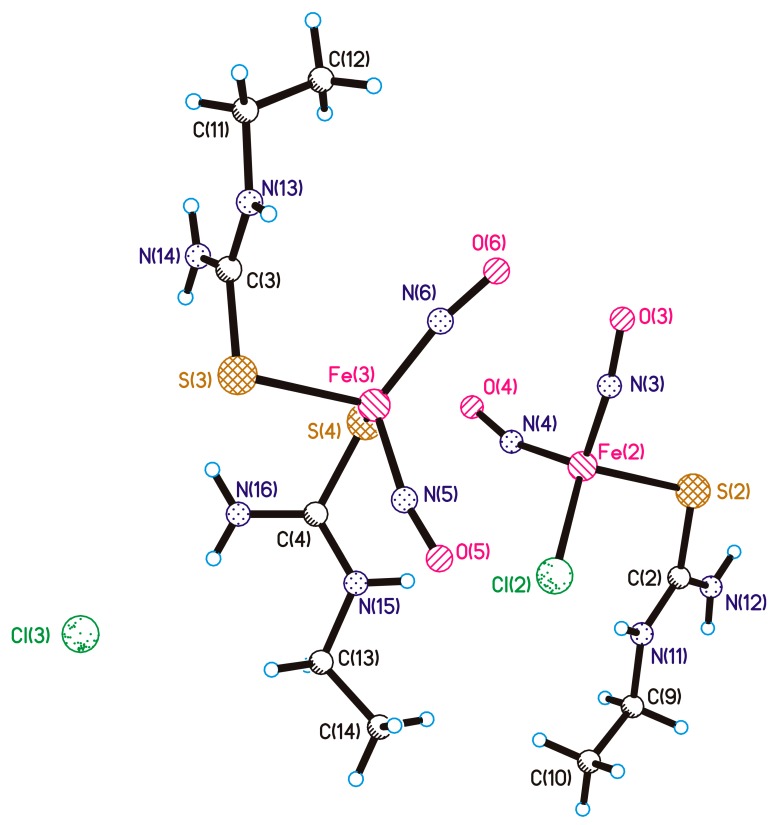
Molecular structure of ETM with the atomic numbering scheme.

Recently, DNIC with *N*-ethylthiourea ligand (ETM, Figure 1) were synthesized, and its structure and NO-donor ability established. Antiproliferative effects of ETM were studied on HeLa and MCF7 cell lines. The complex inhibited proliferation of HeLa cells but had little effect on MCF7 cells [26].

In this study, we examined the antiproliferative effects of ETM in a range of cell lines of different origin: breast, colon, brain, epidermoid, pancreatic, hepatic tumor, and non-cancerous Vero cells.

## 2. Results

### Effects of ETM on Еру Viability of Cells of Different Origin

Toxicity of ETM was studied on cell lines originated from non-cancerous epithelium (Vero cell line) or from different tumors: ER-negative and ER-positive breast carcinomas BT-20 and BT-474, breast carcinosarcoma Hs578T, pancreas carcinoma PANC-1, colon carcinoma Caco2, epidermoid carcinoma A431 and glioma A172 cell lines.

After 72 h exposure ETM exhibited little toxicity to most cell lines studied (Table 1) with IC_50_ values ranging from 200 μM for BT-474 cells to 640 μM for PANC-1 cells. Within this range, cell lines originated from tumors of breast (BT-474, Hs578T) and colon (Caco2) are more susceptible to ETM, while other tumor cells (epidermoid A431, breast BT-20, hepatic HepG2 and pancreatic PANC-1 cells) are less sensitive to ETM than non-cancerous Vero cells. On the other hand, ETM is highly toxic to glioma A172 cells with the IC_50_ approximately three degrees of magnitude lower than that for other cell lines.

**Figure 2:**
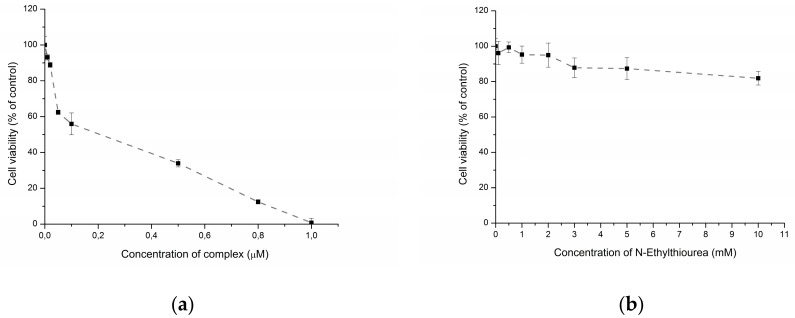
Cytotoxicity towards A172 cells of ETM (**a**) and *N*-ethylthiourea (**b**) after 72 h exposure.

Decomposition of DNICs results in the release of NO and thiolates. To find out if the observed toxic effect of ETM was conferred by its ligand or NO, the cytotoxicity of *N*-ethylthiourea was studied. At concentrations of up to 10 mM, *N*-ethylthiourea exerted very low toxicity towards A172 cells (Figure 2), thus suggesting that the effect ETM can be attributed to its NO-donor ability.

**Figure 3:**
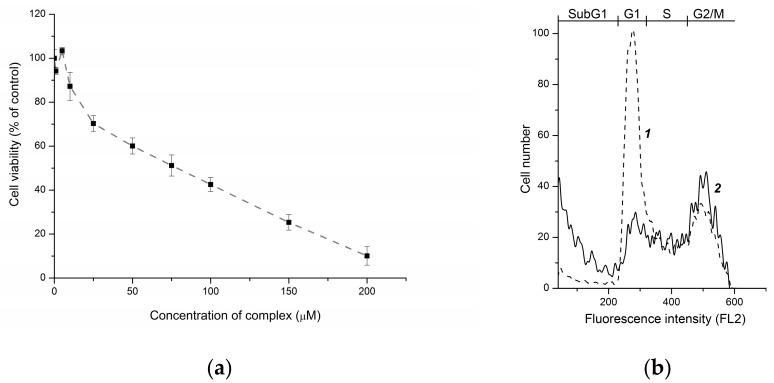
Cell viability inhibition (**a**) and cell cycle changes (**b**) caused by ETM in A172 cells after 24 h exposure. A, the dose-effect curve. B, the histograms of propidium iodide DNA staining in the untreated (1) and ETM-treated cells (2); on the top, the cell cycle phases, as well as cell population with decreased DNA content (SubG1, dying cells) are marked.

Flow cytometry analysis revealed that ETM can induce cell death in A172 cells. The cell cycle was assessed after 24 h exposure since growth of cells for a long time leads to changes in the cell cycle—even in untreated cells—that occur when the culture reaches high density. The IC_50_ for ETM after 24 h is approximately 50 μM (Figure 3a). When used at this concentration, ETM caused cell cycle perturbation and cell death: a decreased number in G1 phase, some increased cell number in G2/M phases, and accumulation of cells in sub-G1 fraction (Figure 3b, Table 2).

Thus, NO-donor complex ETM specifically suppresses the viability of cells in the glioma A172 cell line, being substantially less toxic towards other tumor cells and non-cancerous Vero cells. ETM can induce cell death in A172 cells. The toxicity to glioma A172 cells is attributed mainly to NO, since the *N*-ethylthiourea ligand generated during the decomposition of ETM is not toxic towards the cells.

## 3. Discussion

NO plays versatile functions in tumor biology, and the possible role of modulating NO in cancer therapeutics have been discussed [7]. We have found that *N*-ethylthiourea-containing dinitrosyl iron complex exerted cytotoxicity towards tumor and normal cells. While the susceptibility of most tumor cell lines to ETM was similar to that of normal cells, the glioma A172 cells were found to be profoundly more sensitive to the complex.

Glioblastoma is the most aggressive and invasive of primary brain tumors in adults [27]. The median survival time after presently used therapy is approximately 15 months [28]; thus, new therapeutics for management of glioblastoma are needed. The NO modulation in tumor cells is considered as one of such approaches.

The antitumor potential of NO has been studied on a number or glioma models. *S*-nitroso-*N*-acetyl-penicillamine (SNAP) and sodium nitroprusside (SNP) belonging to *S*-nitrosothiol and metal-NO complex classes of NO donors, inhibited the growth of human glioma cells T98G and U87, and rat glioma cells C6, respectively [29]. NO donor of diazeniumdiolate class, PAPA NONOate, inhibited proliferation of C6 glioma cells [30]. Glycosylated NONOate β-galactosyl-pyrrolidinyl diazeniumdiolate induced cell death in rat glioma cell line 9L [31]. SNP inhibited the viability of C6 and human glioma U251 cells. *S*-nitrosoglutathione (GSNO, another representative of *S*-nitrosothiol class of NO donors) also exerted inhibiting effect on U251 cells. SNP induced generation of reactive oxygen species and apoptosis in C6 cells [32]. SNAP exerted cytotoxic effect on T98G cells. The cytotoxicity of SNAP towards T98G cells was greater than in human osteosarcoma 143B cells. The glucose conjugated SNAP (2-gluSNAP) was substantially (appr. 100-fold) more toxic towards T98G cells compared to SNAP [33]. Diazeniumdiolates diethylamine NONOate (DEA/NO) and spermine NONOate (SPER/NO) but not proline NONOate (PROLI/NO) inhibited the viability of C6 cells. SPER/NO produced similar toxic effects in C6 and U87 cells, primary glioblastoma multiforme cell line, as well as in non-neoplastic human astrocytes and fibroblasts. SPER/NO induced apoptosis in C6 cells, astrocytes and fibroblasts; its pro-apoptotic effect was more prominent in C6 cells than in astrocytes and fibroblasts after 24 h but not 48 h of exposure [34]. Safdar and Taite reported the synthesis of glioma-specific NO donors, diazeniumdiolate derivatives of chlorotoxin, and synthetic 21-mer oligopeptides that have been shown to have high affinity to glioma cells. The two NO donors bound preferentially to glioma T98G and U87 cells and showed minimal binding to non-cancerous cells, normal human astrocytes and human brain microvascular endothelial cells [35].

Many different of mechanisms have been proposed to underlie the cytotoxic effects of NO [6]. Among the others, the multifunctional transcription factor NF-κB is of a particular interest. Over the past decade, NF-κB has emerged as an important factor of cancer development and progression that may be useful targets for cancer treatment [36]. In particular, NF-κB is considered to be a therapeutic target in gliomas [37,38].

NO may inhibit NF-κB through two main mechanisms: the NF-κB inhibitor IκB is stabilization through *S*-nitrosylation, and *S*-nitrosylation of NF-κB which inhibits its DNA binding with further downstream inhibition of anti-apoptotic genes [39,40]. Recently, we have shown that RRE with cysteamine ligand caused *S*-nitrosylation of p65 subunit of NF-κB [25], and impairs its intracellular trafficking and NF-κB DNA binding.

The results of the present work demonstrate that NO-donor dinitrosyl iron complex ETM exhibits pronounced selective cytotoxicity towards glioblastoma cells A172. To our knowledge, this is the first report of a more pronounced susceptibility of glioma cells to a NO donor compared to other tumor cells. In the paper by Subbarayan and coworkers [33] mentioned above, the toxicity of SNAP was higher towards glioblastoma compared to osteosarcoma cells, based on the authors’ Figure 1. Among glioblastoma cell lines, A172 cells have been shown to be the most resistant to NO donor SNP: while the EC_50_ values after 16 h exposure for C6, LN-229, T98G and LN-18 cells were close to 1 mM, EC_50_ for A172 cells was approximately 3 mM [41]. Thus, one could speculate that different glioma cell lines could be similarly susceptible to other spontaneously decomposed NO donors. If the higher sensitivity to NO donors is common among glioma cells, this should be the subject of further investigation, but the just-mentioned data provide some as-yet circumstantial evidence in support of the selective toxicity of NO donors.

The pivotal question to be addressed is the mechanism that accounts for the observed phenomenon of selective toxicity of ETM (and, probably, other compounds of, at least, the DINC class of NO donors). This is also the subject for further investigation.

## 4. Materials and Methods

### 4.1. Synthesis

Complex ETM was synthesized by method [26].

### 4.2. Cell Culture

Vero, A431, PANC-1, A172 cells were cultured in DMEM (PanEco, Moscow, Russia). BT-20, HepG2, Hs578T cells were cultured in EMEM (PanEco, Russia). BT-474 cells were cultured in RPMI (PanEco, Russia). All cell lines were cultured at 37 °C under 5% CO_2_ in the presence of 10% fetal calf serum (BioWest, France), penicillin/streptomycin (PanEco, Russia). BT-474 and Hs578T cells were maintained in medium supplemented with 1 μg/mL insulin (PanEco, Russia). All cell lines were obtained from the All-Russian Collection of Vertebrate Cell Cultures (Institute of Cytology RAS, St.-Petersburg, Russia).

### 4.3. Cytotoxicity Studies and Determination of IC_50_ Doses

For all experiments, ETM and *N*-ethylthiourea were dissolved in 400 μM Tris-HCl buffer (pH 7.5). The final concentration of Tris-HCl in all samples was 40 μM. Cytotoxicity was studied using the MMT test. The cells were plated in 96-well plates (5 × 10^3^ cells per well) in standard incubation media. 24 h after plating, cells were exposed to ETM or *N*-ethylthiourea for 72 h, then 3-(4,5-dimethylthiazol-2-yl)-2,5-diphenyltetrazolium bromide (MTT) was added to cells at the final concentration of 0.45 mg·mL^−1^, and cells incubated in the presence of MTT for more 4 h. After incubation, medium was discarded, and formazan crystals were dissolved in 100 μL DMSO. The staining intensity of reduced formazan was determined with photometry at wavelength of 570 nm. The intensity of MTT staining of cells treated with Tris-HCl was taken as 100%. The IC_50_ values were calculated using the median effect analysis [42].

### 4.4. Flow Cytometry

The A172 cells were plated in 6-well plates in the standard incubation medium (4 × 10^5^ cells per well). 24 h after the plating, cells were exposed to ETM at concentration of 50 μM. To study the cell cycle, cells were collected by trypsinization, washed three times with PBS (pH 7.4), and then fixed and permeabilized by a dropwise addition of 70% aqueous ethanol precooled to −20 °C. The samples were kept for 4 h at +4 °C. Ethanol was removed from fixed samples by washing with PBS, then cells were resuspended in PI staining solution (0.01 mg·mL^−1^ propidium iodide and 0.1 mg·mL^−1^ RNase A in PBS) and incubated at room temperature for 30 min [43]. The samples were subjected to flow cytometry on a Guava easyCyte System (Millipore, Hayward, CA, USA) with Guava^®^ Cell Cycle Assay software (guavaSoft™ 3.1.1, Millipore, Hayward, CA, USA ). Fluorescence of propidium iodide was detected with a 488 nm excitation laser and a 695 nm emission filter.

### 4.5. Statistical Analysis

All data are expressed as mean ± SD of three independent experiments. Statistical significance was analyzed using Student’s *t*-test. The criterion of statistical significance was * *p* < 0.01; ** *p* < 0.001.

## 5. Conclusions

This study described, for the first time, the cytotoxic effects of NO-donor iron nitrosyl complex with *N*-ethylthiourea ligand in normal and different tumor cells. The complex specifically inhibited the viability of glioma A172 cells, while having little effect on cells from breast, skin, colon, hepatic and pancreatic tumors as well as on normal cells.

## Figures and Tables

**Table 1:** Toxicity of ETM to normal and tumor cell lines.

Cell Line	Mean IC_50_ ± SD (µM)
Vero	359.80 ± 14.94
A431	412.84 ± 35.76
BT-20	393.62 ± 10.56
BT-474	202.71 ± 9.25 **
Hs578T	275.30 ± 22.88 *
HepG2	356.07 ± 8.30
Caco2	240.89 ± 5.43 **
A172	0.41 ± 0.15 **
PANC-1	638.95 ± 17.62 **

* *p* < 0.01; ** *p* < 0.001 versus Vero cells.

**Table 2:** Cell cycle distribution of A172 cells after treatment with ETM.

	SubG1	G1	S	G2/M
**control**	5.72%	48.54%	21.90%	23.84%
**ETM, 50 μM, 24 h**	30.90%	15.08%	21.71%	32.31%

## References

[1] Ghimire K., Altmann H.M., Straub A.C., Isenberg J.S. (2017). Nitric oxide: What’s new to NO?. Am. J. Physiol. Cell Physiol..

[2] Thomas D.D., Ridnour L.A., Isenberg J.S., Flores-Santana W., Switzer C.H., Donzelli S., Hussain P., Vecoli C., Paolocci N., Ambs S. (2008). The chemical biology of nitric oxide: Implications in cellular signaling. Free Radic. Biol. Med..

[3] Cheng H., Wang L., Mollica M., Re A.T., Wu S., Zuo L. (2014). Nitric oxide in cancer metastasis. Cancer Lett..

[4] Burke A.J., Sullivan F.J., Giles F.J., Glynn S.A. (2013). The yin and yang of nitric oxide in cancer progression. Carcinogenesis.

[5] Hirst D., Robson T. (2010). Nitric oxide in cancer therapeutics: Interaction with cytotoxic chemotherapy. Curr. Pharm. Des..

[6] Bonavida B., Garban H. (2015). Nitric oxide-mediated sensitization of resistant tumor cells to apoptosis by chemo-immunotherapeutics. Redox Biol..

[7] Huerta S., Chilka S., Bonavida B. (2008). Nitric oxide donors: Novel cancer therapeutics (review). Int. J. Oncol..

[8] Mülsch A., Mordvintcev P., Vanin A.F., Busse R. (1991). The potent vasodilating and guanylyl cyclase activating dinitrosyl-iron (II) complex is stored in a protein-bound form in vascular tissue and is released by thiols. FEBS Lett..

[9] Richardson D.R., Lok H.C. (2008). The nitric oxide-iron interplay in mammalian cells: Transport and storage of dinitrosyl iron complexes. Biochim. Biophys. Acta.

[10] Lewandowska H. (2013). Coordination Chemistry of Nitrosyls and Its Biochemical Implications. Struct. Bond..

[11] Borodulin R.R., Kubrina L.N., Mikoyan V.D., Poltorakov A.P., Shvydkiy V.O., Burbaev D.S., Serezhenkov V.A., Yakhontova E.R., Vanin A.F. (2013). Dinitrosyl iron complexes with glutathione as NO and NO(+) donors. Nitric Oxide.

[12] Keszler A., Diers A.R., Ding Z., Hogg N. (2017). Thiolate-based dinitrosyl iron complexes: Decomposition and detection and differentiation from *S*-nitrosothiols. Nitric Oxide.

[13] Feelisch M., te Poel M., Zamora R., Deussen A., Moncada S. (1994). Understanding the controversy over the identity of EDRF. Nature.

[14] Vanin A.F., Stukan R.A., Manukhina E.B. (1996). Physical properties of dinitrosyl iron complexes with thiol-containing ligands in relation with their vasodilator activity. Biochim. Biophys. Acta.

[15] Lobysheva I.I., Stupakova M.V., Mikoyan V.D., Vasilieva S.V., Vanin A.F. (1999). Induction of the SOS DNA repair response in Escherichia coli by nitric oxide donating agents: Dinitrosyl iron complexes with thiol-containing ligands and *S*-nitrosothiols. FEBS Lett..

[16] Vasilieva S.V., Moshkovskaya E.Y., Sanina N.A., Aldoshin S.M., Vanin A.F. (2004). Genetic signal transduction by nitrosyl-iron complexes in Escherichia coli. Biochemistry.

[17] Kleschyov A.L., Strand S., Schmitt S., Gottfried D., Skatchkov M., Sjakste N., Daiber A., Umansky V., Munzel T. (2006). Dinitrosyl-iron triggers apoptosis in Jurkat cells despite overexpression of Bcl-2. Free Radic. Biol. Med..

[18] Chen T.N., Lo F.C., Tsai M.L., Shih K.N., Chiang M.H., Lee G.H., Liaw W.F. (2006). Dinitrosyl iron complexes [E_5_Fe(NO)_2_]^−^ (E = S, Se): A precursor of Roussin’s black salt [Fe_4_E_3_(NO)_7_]^−^. Inorg. Chim. Acta.

[19] Wen Y.D., Ho Y.L., Shiau R.J., Yeh J.K., Wu J.Y., Wang W.L., Chiou S.J. (2010). Synergistic antitumor effect of curcumin and dinitrosyl iron complexes for against melanoma cells. J. Organomet. Chem..

[20] Shiau R.J., Wu J.Y., Chiou S.J., Wen Y.D. (2012). Effects of curcumin on nitrosyl-iron complex-mediated DNA cleavage and cytotoxicity. Planta Med..

[21] Giliano N.Y., Konevega L.V., Noskin L.A., Serezhenkov V.A., Poltorakov A.P., Vanin A.F. (2011). Dinitrosyl iron complexes with thiol-containing ligands and apoptosis: Studies with HeLa cell cultures. Nitric Oxide.

[22] Wu S.C., Lu C.Y., Chen Y.L., Lo F.C., Wang T.Y., Chen Y.J., Liaw W.F., Wang Y.M. (2016). Water-Soluble Dinitrosyl Iron Complex (DNIC): A Nitric Oxide Vehicle Triggering Cancer Cell Death via Apoptosis. Inorg. Chem..

[23] Stupina T.S., Parkhomenko I.I., Balalaeva I.V., Kostyuk G.V., Sanina N.A., Terent´ev A.A. (2011). Cytotoxic properties of the nitrosyl iron complex with phenylthiyl. Russ. Chem. Bull. Int. Ed..

[24] Sanina N.A., Kozub G.I., Zhukova O.S., Emel’yanova N.S., Kondrat’eva T.A., Korchagin D.V., Shilov G.V., Ovanesyan N.S., Aldoshin S.M. (2013). Synthesis, structure, NO donor activity of iron–sulfur nitrosyl complex with 2-aminophenol-2-yl and its antiproliferative activity against human cancer cells. J. Coord. Chem..

[25] Stupina N.S., Terent’ev A.A., Antonova N.O., Balalaeva I.V., Sanina N.A., Aldoshin S.M. (2014). Influence of sulfur-nitrosyl iron complexes of “µ-S” structural type on NF-κB nuclear factor. Int. Sci. J. Med. Biol. Sci..

[26] Sanina N.A., Shmatko N.Y., Korchagin D.V., Shilov G.V., Terent’ev A.A., Stupina T.S., Balakina A.A., Komleva N.V., Ovanesyan N.S., Kulikov A.V. (2016). A New member of the cationic dinitrosyl iron complexes family incorporating *N*-ethylthiourea is effective against human HeLa and MCF-7 tumor cell lines. J. Coord. Chem..

[27] Ostrom Q.T., Gittleman H., Liao P., Rouse C., Chen Y., Dowling J., Wolinsky Y., Kruchko C., Barnholtz-Sloan J. (2014). CBTRUS statistical report: Primary brain and central nervous system tumors diagnosed in the United States in 2007–2011. Neuro Oncol..

[28] Vakilian A., Khorramdelazad H., Heidari P., Rezaei Z.S., Hassanshahi G. (2017). CCL2/CCR2 signaling pathway in glioblastoma multiforme. Neurochem. Int..

[29] Kurimoto M., Endo S., Hirashima Y., Hamada H., Ogiichi T., Takaku A. (1999). Growth inhibition and radiosensitization of cultured glioma cells by nitric oxide generating agents. J. Neurooncol..

[30] Viani P., Giussani P., Brioschi L., Bassi R., Anelli V., Tettamanti G., Riboni L. (2003). Ceramide in nitric oxide inhibition of glioma cell growth. Evidence for the involvement of ceramide traffic. J. Biol. Chem..

[31] Chen C., Shi Y., Li S., Qi Q., Gu L., Song J., Wang P.G. (2006). A glycosylated nitric oxide donor, beta-Gal-NONOate, and its site-specific antitumor activity. Arch. Pharm. (Weinheim).

[32] Janjetovic K., Misirkic M., Vucicevic L., Harhaji L., Trajkovic V. (2008). Synergistic antiglioma action of hyperthermia and nitric oxide. Eur. J. Pharmacol..

[33] Subbarayan P.R., Wang P.G., Lampidis T.J., Ardalan B., Braunschweiger P. (2008). Differential expression of Glut 1 mRNA and protein levels correlates with increased sensitivity to the glyco-conjugated nitric oxide donor (2-glu-SNAP) in different tumor cell types. J. Chemother..

[34] Weyerbrock A., Baumer B., Papazoglou A. (2009). Growth inhibition and chemosensitization of exogenous nitric oxide released from NONOates in glioma cells in vitro. J. Neurosurg..

[35] Safdar S., Taite L.J. (2012). Targeted diazeniumdiolates: Localized nitric oxide release from glioma-specific peptides and proteins. Int. J. Pharm..

[36] Li F., Zhang J., Arfuso F., Chinnathambi A., Zayed M.E., Alharbi S.A., Kumar A.P., Ahn K.S., Sethi G. (2015). NF-κB in cancer therapy. Arch. Toxicol..

[37] Atkinson G.P., Nozell S.E., Benveniste E.T. (2010). NF-κB and STAT3 signaling in glioma: Targets for future therapies. Expert Rev. Neurother..

[38] Puliyappadamba V.T., Hatanpaa K.J., Chakraborty S., Habib A.A. (2014). The role of NF-κB in the pathogenesis of glioma. Mol. Cell. Oncol..

[39] Marshall H.E., Hess D.T., Stamler J.S. (2004). *S*-nitrosylation: Physiological regulation of NF-κB. Proc. Natl. Acad. Sci. USA.

[40] Colasanti M., Persichini T. (2000). Nitric oxide: An inhibitor of NF-μB/Rel system in glial cells. Brain Res. Bull..

[41] Rieger J., Ständer M., Löschmann P.A., Heneka M., Dichgans J., Klockgether T., Weller M. (1998). Synthesis and biological effects of NO in malignant glioma cells: Modulation by cytokines including CD95L and TGF-β, dexamethasone, and p53 gene transfer. Oncogene.

[42] Chou T.C., Talalay P. (1984). Quantitative analysis of dose-effect relationships: The combined effects of multiple drugs or enzyme inhibitors. Adv. Enzyme Regul..

[43] Pozarowski P., Darzynkiewicz Z. (2004). Analysis of cell cycle by flow cytometry. Methods Mol. Biol..

